# Bacterial Adhesion of *Streptococcus suis* to Host Cells and Its Inhibition by Carbohydrate Ligands

**DOI:** 10.3390/biology2030918

**Published:** 2013-07-01

**Authors:** Annika Kouki, Roland J. Pieters, Ulf J. Nilsson, Vuokko Loimaranta, Jukka Finne, Sauli Haataja

**Affiliations:** 1 Department of Medical Biochemistry and Genetics, University of Turku, Kiinamyllynkatu 10, Turku FI-20520, Finland; E-Mails: annika.kouki@utu.fi (A.K.); vuokko.loimaranta@utu.fi (V.L.); 2 Department of Medicinal Chemistry and Chemical Biology, Utrecht Institute for Pharmaceutical Sciences, Utrecht University, P.O. Box 80082, Utrecht 3508 TB, The Netherlands; E-Mail: r.j.pieters@uu.nl; 3 Centre for Analysis and Synthesis, Department of Chemistry, Lund University, POB 124, Lund SE-22100, Sweden; E-Mail: ulf.nilsson@chem.lu.se; 4 Department of Biosciences, Division of Biochemistry and Biotechnology, University of Helsinki, P.O.B. 56, Helsinki FI-00014, Finland; E-Mail: jukka.finne@helsinki.fi

**Keywords:** bacterial adhesion, galabiose, virulence, *Streptococcus suis*, carbohydrate, adhesin, Gb3, galactose

## Abstract

*Streptococcus suis* is a Gram-positive bacterium, which causes sepsis and meningitis in pigs and humans. This review examines the role of known *S. suis* virulence factors in adhesion and *S. suis* carbohydrate-based adhesion mechanisms, as well as the inhibition of *S. suis* adhesion by anti-adhesion compounds in *in vitro* assays. Carbohydrate-binding specificities of *S. suis* have been identified, and these studies have shown that many strains recognize Galα1-4Gal-containing oligosaccharides present in host glycolipids. In the era of increasing antibiotic resistance, new means to treat infections are needed. Since microbial adhesion to carbohydrates is important to establish disease, compounds blocking adhesion could be an alternative to antibiotics. The use of oligosaccharides as drugs is generally hampered by their relatively low affinity (micromolar) to compete with multivalent binding to host receptors. However, screening of a library of chemically modified Galα1-4Gal derivatives has identified compounds that inhibit *S. suis* adhesion in nanomolar range. Also, design of multivalent Galα1-4Gal-containing dendrimers has resulted in a significant increase of the inhibitory potency of the disaccharide. The *S. suis* adhesin binding to Galα1-4Gal-oligosaccharides, Streptococcal adhesin P (SadP), was recently identified. It has a Galα1-4Gal-binding *N*-terminal domain and a *C*-terminal LPNTG-motif for cell wall anchoring. The carbohydrate-binding domain has no homology to *E. coli* P fimbrial adhesin, which suggests that these Gram-positive and Gram-negative bacterial adhesins recognizing the same receptor have evolved by convergent evolution. SadP adhesin may represent a promising target for the design of anti-adhesion ligands for the prevention and treatment of *S. suis* infections.

## 1. Introduction

*Streptococcus suis* is a Gram-positive bacterium, which is an emerging cause of serious infections, such as meningitis, septicemia, endocarditis and pneumonia in pigs and zoonotic meningitis in humans [1,2,3,4]. There are currently 35 known capsular serotypes in *S. suis*, of which the serotype 2 capsule (SS2) is the most virulent. Recently, multilocus sequencing has been used to more accurately identify the relatedness of virulent strains. Based on this, *S. suis* sequence type 1 and 7 (ST1 and ST7) have been found to be associated with the most severe infections [5].

Host- and tissue-specific adhesion of both the Gram-negative and Gram-positive bacteria is a prerequisite for infection and invasive disease [6,7]. Bacteria interact with host mucosal cells in order to colonize their ecological niche. Adhesion is important for avoiding the cleaning mechanisms of the host mucociliary system. Bacteria have evolved multiple adhesins to specifically recognize host cell surface carbohydrate and protein receptors [8]. 

*S. suis* has rapidly evolved drug resistance against antibiotics by horizontal transfer [9]. Therefore, novel means to prevent and treat infections are needed. Anti-adhesion therapy is based on the inhibition of bacterial attachment to a specific receptor structure [10,11]. Once the structures of the receptors are known, more potent high-affinity receptor analogs can be designed. An advantage of the prevention of infections by receptor analogs could be that bacteria do not develop resistance, in contrast to the traditional bactericidal drugs. Since anti-adhesive compounds target adhesins, which are required for a specific colonization of the host, the mutant phenotypes of bacteria deficient in adhesion would be eliminated by the host. 

Previous studies have shed new light on how *S. suis* interacts with host cells. In many cases, virulence genes are involved directly or indirectly with adhesion. In this review, virulence mechanisms with a role in adhesion are discussed. Particularly, *S. suis* adhesion mechanisms that are based on the recognition of carbohydrate receptors, as well as the development of carbohydrate-based anti-adhesive compounds are reviewed. 

## 2. *Streptococcus suis* Adhesion

*Streptococcus suis* of the capsular polysaccharide type 2 is globally the most common isolate from both the porcine and human cases of meningitis [12]. It is typically isolated from the nasal cavity and tonsils of the pigs, which can carry this bacterium asymptomatically for a long period of time. The exact mechanisms, which trigger the infectious disease and by which *S. suis* invades the blood circulation, are not known. It is thought that *S. suis* colonizing the upper respiratory tract may invade through the respiratory epithelium or via the intestine [13]. Once the bacteria have reached the blood circulation, they may penetrate into the brain by adhering to brain capillary endothelial cells and cross the blood brain barrier. The molecular mechanisms of *S. suis* interaction with different tissue compartments and cells are poorly known.

*S. suis* adhesion and invasion to the cultured epithelial cells commonly used in laboratories, such as A549, HeLa, MDCK and Hep-2 cells, has been studied. A polysaccharide capsule has been found to partially mask the adhesion [14]. The unencapsulated serotype 2 and non-typeable strains have been found to be more adhesive and invasive than encapsulated strains [15]. The invasion mechanisms of *S. suis* into host cells have been suggested to be closely related to other *Streptococci*. *S. suis* was described upon interaction with the host cell membrane to form large membrane invaginations or filopodia-like protrusions, followed by a process called close-contact-induced membrane-triggering [15].

Adhesion to the capillary endothelial cells precedes the penetration of *S. suis* into the brain from blood circulation and could be mediated by either direct invasion of bacteria into the cells by endocytic mechanisms or by disrupting the cell junctions. The adhesion of *S. suis* to the brain microvascular cells has been reported to be independent of the capsular polysaccharide expression. *S. suis* type 2 strains have been found to adhere to both human and porcine microvascular endothelial cells, but to invade only porcine cells with actin-dependent mechanisms [16]. Recently, *S. suis* and *Neisseria meningitidis* have been found to invade into the human choroid plexus papilloma cells and cross from the basolateral onto the apical side, thus modelling bacterial entry from the blood into the cerebrospinal side [17,18]. *S. suis* can also bind and activate plasminogen to proteolytically make the blood-brain barrier leaky to bacteria [19]. Furthermore, the *S. suis* polysaccharide capsule and cell wall can synergistically induce production of prostaglandin and metalloproteinase by macrophages and play a critical role in the permeabilization of the blood-brain barrier [20].

## 3. Virulence Genes with a Role in Adhesion

### 3.1. Regulators

During the colonization of the upper respiratory tract and the invasion of the blood circulation, *S. suis* needs to regulate the expression of the proteins involved in adhesion. Recent evidence shows that regulators required for *in vivo* virulence (Table 1) also modulate adhesion properties.

The regulatory genes required for virulence have been characterized by the comparison of the knockout mutants and wild-type bacteria for their survival both in mice and in pig infection models. Several two-component regulatory systems (TCS) regulate gene expression in *Streptococci*, such as those required for the natural competence of genetic transformation and quorum sensing [21,22]. TCS consist of a sensor kinase protein, which phosphorylates and activates a transcriptional regulator that binds to promoters to induce or repress the expression of a large number of genes [23]. An orphan two-component transcriptional regulator can also control the transcription of virulence genes. Knockout strains harboring deletion in the *S. suis ciaRH* [24] and in the genes *revS* and *revSC21* encoding orphan regulators show decreased adhesion to Hep-2 cells [25,26]. SalK/SalR is another recently described TCS required for the virulence of *S. suis* in the porcine model, but its role in the regulation of adhesion is not well known [27,28]. The two-component system CovR/S regulates a number of *S. pyogenes* virulence genes. *S. suis* has an orphan orthologue of *covR*, also known as *csrR* (capsule synthesis regulator), that was found to negatively modulate virulence [29]. *S. pyogenes* Rgg, also known as RopB, is a transcriptional regulator known to regulate the expression of dozens of genes and has been shown to repress adhesion to epithelial cells and negatively regulate virulence in a murine intraperitoneal model [30,31,32,33]. In *S. suis*, the genes, *covR* and *rgg*, are required for virulence, and the corresponding knockout strains show increased binding to Hep-2 cells [29,34].

**Table 1:** *S. suis* virulence genes with known phenotype in adhesion or interaction with host cells. The chromosomal locations of the genes in the genome of *S. suis* type 2 P1/7, **GZ1** or **ST1** [35] are indicated.

GENE ID	GENE DESIGNATION	MUTANT PHENOTYPES
Regulators with phenotype in adhesion		
SSU0944, SSU0945	*ciaRH* [24]	Two-component regulator, decreased adhesion to Hep-2 cells
SSU1873 not functional in P1/7, SSGZ1_1897 in *S. suis* GZ1	*revS* [25]	Orphan regulator, decreased adhesion to Hep-2 cells
–	*revSC21* [26]	Orphan regulator, decreased binding to Hep-2 cells
SSU0376	*luxS* [36]	Quorum sensing regulator, decreased adhesion
SSU1789	*rgg-like regulator* [34]	Negative transcriptional regulator, increased adhesion to Hep-2 cells
SSU1191	*covR* [29]	Orphan regulator, increased adhesion to Hep-2 cells
SSU1202	*ccpA* [37]	Carbon catabolite protein, decreased capsule thickness
Modulators of adhesion		
SSU0516, SSU0519, SSU0520, SSU0517, SSU0535	*cps2B*, *cps2E*, *cps2F* [15,38], *cps2C* [39], *neuB* [39]	Polysaccharide synthesis genes, increased adhesion of unencapsulated mutants
SSU0596, SSU1448	*dltA and pgdA* [40,41]	Cell wall modification, upregulated upon contact with endothelial cells
Moonlighting or other cell wall proteins without signal sequence and known anchoring mechanism		
SSU0187, SSGZ1_0184	*dpp4* [42]	Dipeptidyl peptidase IV, fibronectin binding
SSU1320	*eno* [43,44]	Enolase, fibronectin and plasminogen binding, recombinant protein inhibits adhesion to Hep-2 cells
SSU0153	*GAPDH*, [45]	Recombinant protein inhibits bacterial binding to porcine tracheal rings and Hep-2 cells
SSU1541	*gnd* [46]	6-Phosphogluconate-dehydrogenase, recombinant protein inhibits bacterial binding to Hep-2 and HeLa cells
SSU0157	*glnA* [47]	Glutamine synthetase, decreased adherence to the Hep-2 cells
SSU1127	*atl*, *autolysin* [48]	Biofilm and Hep-2 cell adhesion
SSU1311	*fbps* [49]	Fibronectin binding
LPXTG-anchored proteins		
SSU0925	*srtA* [50]	Anchoring of cell wall proteins
SSU0879	*IgA1 protease* [51,52]	Degradation of IgA protecting mucosal surfaces
SSU1474 (pseudo), SSUST1_ 1540 in *S. suis* ST1	*sof* [53]	Lipoprotein degradation
SSU0757	*sspA*, [54,55]	Subtilisin-like protease, induces secretion of cytokines and chemokines
SSU1143	*ssa* [56]	Fibronectin/fibrinogen binding, reduced adhesion and invasion to Hep-2 cells

An autoinducer AI-2/LuxS quorum sensing system regulates virulence and biofilm formation in *Streptococci* [57,58]. In *S. suis*, it positively regulates biofilm formation and adhesion to Hep-2 cells [36]. In addition, a deletion mutant in the gene, *stp*, which encodes a serine/threonine phosphatase, has been shown to adhere less to Hep-2 cells compared to the wild-type strain [59]. A global regulatory protein, called catabolite control protein, CcpA, regulates the utilization of carbohydrates by *S. suis*. It also regulates polysaccharide capsule thickness and hemolytic activity [37]. The regulation of *S. suis* capsule thickness might affect the adhesion properties. Conclusively, in *S. suis*, a complex network of regulation of virulence also modulates adhesion.

### 3.2. S. suis Surface Glycoconjugates

The *S. suis* capsular polysaccharide can block the adhesins at the cell surface. Unencapsulated strains agglutinate erythrocytes and adhere more strongly to HEp-2 cells than encapsulated strains [15,60]. The serotype 2 polysaccharide structure consists of the repeating unit [(Neu5Acα2-6Galβ1-4GlcNAcβ1-3)Galβ1-4(Galα1-3)Rhaβ1-4Glcβ1-]_n_ [61]. Presence of sialic acid in *S. suis* capsular polysaccharide creates a negative charge on the bacterial surface. Therefore, it is likely that a host cell surface negative charge and the sialic acid containing bacterial polysaccharide form a biophysical repulsion strong enough to reduce adhesion of encapsulated *S. suis* to host cells. Recently, mutant strains with the deletion of genes required for serotype 2 capsular polysaccharide synthesis were found to be avirulent in animal models, but more adherent and invasive to Hep-2 cells [39].

*Streptococcus agalactiae* polysaccharide contains sialic acid and is important for the evasion of the complement and phagocytosis, but in the case of *S. suis*, it has been suggested that, due to the lower amount of sialic acid in *S. suis* type 2 capsule, it does not have a major role in the evasion of the complement [61]. Instead, the polysaccharide capsule of type 2 was previously described to be involved in the interaction of *S. suis* with macrophages. Specifically, removal of *S. suis* sialic acid reduced its binding to J774 macrophages [62]. The ligands recognizing sialic acid are not known. The *S. suis* type 2 capsular polysaccharide was recently found to prevent phagocytosis of *S. suis* by destabilizing lipid microdomains of phagocytes. The capsular polysaccharide prevented lactosylceramide accumulation into the phagocytic cup and the activation of lactosylceramide-lipid raft signaling pathways, thus inhibiting the activation of the phosphoinositide 3-kinase/Akt and p38 MAPK pathways required for the activation of phagocytosis [63]. The ability of *S. suis* to adhere to immune cells and to prevent phagocytosis could be a mechanism for the bacteria to cross the blood-brain-barrier, as suggested by the “modified Trojan horse” theory [63].

The role of the *S. suis* polysaccharide capsule in the invasion of bacteria into the brain has been studied in an *in vitro* model of neutrophil transmigration. The neutrophils infected with *S. suis* transmigrated through the choroid plexus epithelial cell monolayer in an inverted Transwell system mimicking the blood-brain barrier [64]. Encapsulated *S. suis* bacteria induced the granulocytes to transmigrate through the porcine choroid plexus epithelial cells from their basolateral sides. This correlates with the theory that the influx of leukocytes into the brain is important for the development of meningitis.

Mutant strains harboring deletions in the alanine-alanine ligase, *dltA*, and peptidoglycan *N*-deacetylase *pgdA* genes, required for modification of lipoteichoic acid and peptidoglycan structure, are more susceptible to the action of antimicrobial peptides and neutrophil killing mechanisms [40,41]. In addition, these genes are required for *in vivo* survival in animal models. Interestingly, the transcription of alanine-alanine ligase and peptidoglycan deacetylase were upregulated upon contact with endothelial cells [65]. It was suggested that the regulation of these genes can prime bacteria to be more resistant for neutrophil killing after they have crossed the blood-brain barrier.

### 3.3. Cell Wall Proteins

Gram-positive bacteria lack the outer membrane, and therefore, proteins either covalently or non-covalently attached to the cell wall have numerous possibilities to interact with host surfaces. The adhesins can be classified into different cell wall-bound protein classes based on their anchor mechanism: proteins that are cytoplasmic and are found in the cell wall fraction; proteins that are non-covalently attached to the cell wall and contain an *N*-terminal sequence for secretion; and proteins that are covalently linked to peptidoglycan and contain an *N*-terminal secretory signal sequence and a *C*-terminal LPXTG-motif [66]. 

*Streptococci* have evolved mechanisms to adhere to the host cells utilizing moonlighting proteins, *i.e.*, cell wall-attached proteins that have enzymatic activities in addition to adhesion specificities [67]. Several *S. suis* enzymes have been reported to be adhesins. Except for interactions with host fibronectin, the host receptors have remained unknown. Fibronectin has been found to be recognized by the *S. suis* cell wall-bound proteins, dipeptidyl peptidase IV, enolase and Fbps [42,43,44,49]. Enolase recognizes also plasminogen. Plasminogen binding, in addition to fibronectin, could cooperatively help to degrade extracellular matrix and fibrin and aid in the invasion of bacteria into tissues. Plasminogen binding seems in many *Streptococci* to be important in invasion [68]. The adhesion properties of moonlighting proteins and their ability to activate host proteases might represent an example of convergent evolution of bacteria to be able to use host proteases in subverting host defense mechanisms. Furthermore, *S. suis* glyceraldehyde-3-phosphate dehydrogenase, 6-phosphogluconate dehydrogenase, glutamine synthetase and autolysin have been identified as moonlighting adhesins [45,46,47,48,69], but their adhesion specificities are not known.

LPXTG-motif containing proteins are covalently anchored to the cell wall peptidoglycan via their *C*-termini. They require a sortase enzyme that covalently attaches them to the peptidoglycan. *S. suis* housekeeping sortase A (SrtA) seems important in adhesion, since a knockout strain was shown to be deficient in the adhesion to endothelial cells [50]. Recently, *srtA* was shown to be required for high virulence and adhesion to Hep-2 cells and human umbilical endothelial cells [70]. Some of the LPXTG-anchored proteins, such as IgA protease, serum opacity factor Sof, subtilisin-like protein SspA and fibronectin-binding protein Ssa are known to be required for virulence [51,52,53,54,55,56]. Several other LPXTG-motif proteins have been described, but relatively little is known of their role in adhesion and virulence (for extensive reviews, see [13,71]).

An important group of proteins containing the LPXTG-anchor motif are pilins, which form the fimbrial structures, called pili, in *Streptococci* [66]. Typical for the fimbriae of Gram-positive bacteria are that the pilins are covalently linked via motifs closely resembling the LPXTG sequences. The linkages are catalyzed by pilus islet-specific sortases. In *S. suis*, the pilus islands srtBCD, srtE, srtF and srtG have been identified [72,73]. The SrtF cluster contains four genes encoding a putative signal peptidase (*siF*), putative ancillary (*sfp2*, pseudogene) and major (*sfp1*) pilus subunits and class C sortase, *srtF* [74]. Surprisingly *sfp2*, which is homologous to other Streptococcal fimbriae-associated adhesins, is a pseudogene, and the experiments carried with WT and *sfb1* mutants indicate that *S. suis* fimbriae are indispensable for attachment to the pig brain capillary endothelial cells and for virulence in mouse sepsis models [74].

Interestingly, studies characterizing gene expression upon bacterial contact with endothelial cells have revealed that the mRNA levels of signal peptidase and sortase E involved in pilus synthesis were increased [65]. This suggests that, with the so far unknown mechanisms, *S. suis* can sense the contact with host cells and induce the expression of genes important for interaction with host cells.

## 4. *S. suis* Carbohydrate-Specific Adhesion to Host Cells

A limited number of *S. suis* carbohydrate-specific adhesion mechanisms have been described. Some *S. suis* strains recognize sialic acid-containing oligosaccharides. Bacteria bind to the terminal sialic acid in polylactosamine chains with the fine specificity of NeuNAcα2-3Galβ1-4GlcNAcβ1-3Gal [75]. Recently, two *S. suis* LPXTG-anchored proteins have been shown to recognize host cell carbohydrates. One of them, protein HP0197, binds host cell surface glycosaminoglycans. The adhesin knockout strain had decreased binding to Hep-2 cells [76]. We have previously identified a Galα1-Gal-binding activity and identified, recently, the corresponding adhesin [77,78]. The binding specificity to Galα1-4Gal (galabiose) and exploitation of receptor analogs in the development of anti-adhesion compounds are discussed below.

### 4.1. Galabiose (Galα1-4Gal)-Specific Adhesion of S. suis

The *S. suis* galabiose-specific adhesion activity was originally characterized with hemagglutination assay based on multivalent recognition of erythrocyte surface carbohydrates by bacterial adhesins [77]. Galα1-4Gal-oligosaccharides are present in globo-series glycolipids, which are abundant in erythrocytes, endothelial and uroepithelial cells. Glycolipids containing the Galα1-4Gal-sequence form the human P blood group system. The *S. suis* galabiose binding strains can be grouped into two groups based on their monosaccharide inhibition pattern in hemagglutination assays [77]. Type P_N_ is inhibited by both galactose and *N*-acetylgalactosamine, whereas type P_O_ is only inhibited by galactose. The *S. suis* hemagglutination is inhibited with oligosaccharides containing the Galα1-4Gal structure at micromolar concentrations, but only at millimolar concentrations by disaccharides containing Galα1-3Gal or Galα1-6Glc linkages [77]. Analysis of the detailed adhesion specificity toward galabiose-containing glycans revealed that it has the highest specificity to globotriaosylceramide GbO3, Galα1-4Galβ1-4Glcβ1-1'Cer (the P^k^ antigen, Table 2).

**Table 2:** Galα1-4Gal-binding proteins ^a^.

Structure/Antigen ^a^		Ligands	
		Adhesins	Toxins
GbO3 ^b^	**Galα1-4Gal**β1-4Glcβ1-1'Cer/P^k^	*E. coli* PapGI [79], *S. suis* SadP, *P. aeruginosa* Lectin I [80]	*S. dysenteriae* Shiga toxin [81], *E. coli* verotoxin 1, 2, 2c [82,83]
GbO4	GalNAcβ1- **3Galα1-4Gal**β1-4Glcβ1-1'Cer/P	*E. coli* PapGII [79]	*E. coli* verotoxin 2e [84]
GbO5	GalNAcα1-3GalNAcβ1- **3Galα1-4Gal**β1-4Glcβ1-1'Cer/Forssman	*E. coli* PapGIII [79]	

^a^ For simplicity, blood group antigen P_1_, **Galα1-4Gal**β1-4GlcNAcβ1-4Galβ1-4Glcβ1-1'Cer, is not included in the table; ^b^ GbO3, GbO4 and GbO5 are abbreviations for globotria-, globotetra- and globopentaosylceramides.

Galα1-4Gal is also recognized by *E. coli* PapG adhesins, *Pseudomonas aeruginosa* lectin I, Shiga toxin and verotoxins (Table 2). The globo-series glycolipid composition varies between animal species, and different galabiose containing glycolipids are host-specifically recognized by uropathogenic *E. coli* P fimbrial adhesins [79] and *E. coli* verotoxins (Table 2).

### 4.2. The Galabiose-Binding Adhesin SadP

The *S. suis* galabiose-specific adhesin has been isolated [78]. The adhesin was extracted from the *S. suis* cell wall with lysozyme and was captured with the pigeon ovomucoid affinity matrix. Pigeon ovomucoid is a strong glycoprotein inhibitor of *S. suis* hemagglutination, as it contains terminal Galα1-4Gal-residues in its oligosaccharides [85,86]. A gene encoding the adhesin was identified as SSU_0253 from the *S. suis* serotype 2 strain P1/7 genomic sequence. Sequence analysis of the gene shows that it has an *N*-terminal signal peptide for secretion, seven *C*-terminal tandem repeats and an LPXTG-motif. An *S. suis sadP* knockout strain lost galabiose binding activity. A recombinant *N*-terminal domain of the adhesin recognizes Galα1-4Gal-oligosaccharides. The adhesin was designated as the Streptococcal adhesin P (SadP).

The SadP galabiose-binding domain has no significant homology to other known galabiose-binding proteins, including *E. coli* P fimbrial adhesins and Shiga toxins and verotoxins (Table 2). An identical *S. suis* protein, Fhb (HP0272), has been reported to bind to human complement factor H, which is a glycoprotein that regulates the complement system [87]. The factor H binding domain is located in the *C*-terminal region, which has homology to *S. agalactiae* IgA-binding protein [78]. The factor H binding protein in *S. suis* strain 05ZYH33 has an *N*-terminal domain that is identical to SadP. The *fhb*-05ZYH33 knockout strain was highly attenuated in a piglet model [87].

**Figure 1:**
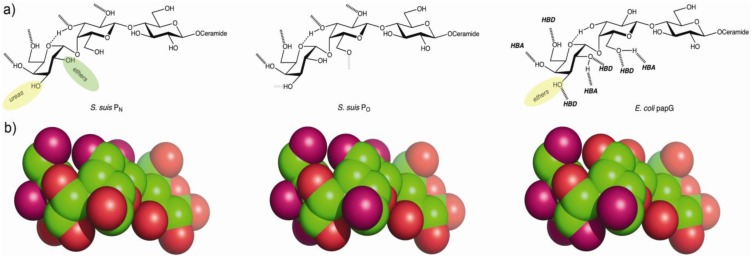
(**a**) Hydrogen bonding patterns of *S. suis* P_N_, *S. suis* P_O_ [77] and *E. coli* PapG adhesins [88,89]. Black dashed lines indicate hydrogen bonds and grey dashed lines indicate possible weak hydrogen bonds. Hydrogen bond directionalities have been mapped for the *E. coli* PapG class I adhesin [90], which was later confirmed by structural analysis of a globotetraose:papG class II adhesin complex [91]. (HBA = hydrogen bond acceptor, HBD = hydrogen bond donor). Derivatization with urea and ether groups at O-3 of α-D-Gal [92] enhances affinity for *S. suis* P_N_ and *E. coli* PapG, respectively (indicated with yellow ovals). Ether groups at O-2 of α-D-Gal enhance binding to *S. suis* P_N_ (indicated with green oval). (**b**) Space-filling models of the globotriose trisaccharide with hydroxyl oxygens interacting with the three adhesins shown in light pink, which illustrates the different epitopes recognized. (Globotriose conformation taken from the globotetraose:papG class II adhesin complex; pdb id 1J8R.)

In the galabiose structure, the essential key hydroxyls for *S. suis* binding are HO-4', HO-6', HO-2 and HO-3 (Figure 1), and in addition, P_O_ strains have weak interactions with HO-3' and HO-6. The combining site of type P_O_ is more narrow on the terminal position of galactose, whereas the P_N_ strains allow substitutions in the HO-3' position. The essential hydroxyls of galabiose required for the binding of *E. coli* PapG adhesins are different. *E. coli* PapG recognizes HO-2', HO-3', HO-4', HO-6' and HO-6 hydroxyls in the disaccharide (Figure 1). The hydrogen bonding pattern suggests that these two bacterial adhesins recognize the disaccharide from different sides [77]. Conclusively, the functional assays with oligosaccharide derivatives and the adhesin sequence comparisons indicate that the galabiose binding *E. coli* PapG and *S. suis* SadP have different modes of mechanisms to interact with Galα1-4Gal and represent an example of convergent evolution of bacterial adhesins toward binding to the same Galα1-4Gal-receptor.

## 5. Towards the Development of Therapy Based on Prevention of Adhesion

Carbohydrates have usually a low affinity to adhesins, partly because the *O*-glycosidic linkages between monosaccharides in oligosaccharides are very flexible. In addition, monovalent oligosaccharide inhibitors are inefficient for competing with the multivalent receptor interactions. Strategies to overcome the relatively low affinity of the lectin-carbohydrate interactions include modification of the hydroxyl groups of the oligosaccharide receptors and construction of multivalent carbohydrate dendrimers to increase the affinity [93,94].

### 5.1. Combinatorial Libraries of Receptor Carbohydrates

Previous data showed that *S. suis* galabiose binding was not dependent on the HO-3' and HO-2' hydroxyls of the terminal α-galactose (Figure 1). The adhesin combining site of type P_N_ allowed larger substituents in the HO-3' position of the terminal galactose, as compared to type P_O_ adhesin. When large libraries of galabiose derivatives were tested for the inhibitory activity with *E. coli* PapG and *S. suis* SadP, galabiosides carrying aromatic structures at C1 were found to be efficient inhibitors of the PapG adhesin [92]. The major difference between PapG and SadP was that methoxymethylation at O2' and phenylurea derivatization at C3' of the terminal galactose in the galabiose disaccharide provided inhibitors of the *S. suis* type P_N_ adhesin with nanomolar affinities.

### 5.2. Dendrimers as Polyvalent Carbohydrate Inhibitors

The mode of interaction between bacterial adhesins of *S. suis* and *E. coli* are different compared to Shiga-like toxins with GbO3 glycolipids. The adhesin can be inhibited with soluble oligosaccharides, whereas Shiga-like toxins are not inhibited and require that the receptor oligosaccharide is presented polyvalently. Utilizing the crystal structure of the AB5 Shiga-like toxin, a “STARFISH” oligomeric water-soluble compound was designed consisting of five trisaccharides at the tip of spacers. It was found to occupy all five receptor binding sites and had subnanomolar inhibitory activity [95]. The inhibition of *S. suis* type P_N_ and P_O_ hemagglutination activities with dendrimeric galabiosides have been studied [96]. The inhibitions were tested under static conditions using hemagglutination in microtiter wells. The relative potencies of the dendrimeric compounds increased to 300–400-fold when galabiose was presented as a divalent compound.

Bacteria adhere often under share stress and in dynamic flow conditions; therefore, a surface plasmon resonance assay has been used to analyze bacterial binding to galabiose-BSA neoglycoprotein under flow conditions [97]. The inhibitory activity of divalent, tetravalent and octavalent galabiose dendrimers were tested (Table 3). The highest relative inhibitory potency of 250 per sugar was obtained with the tetravalent compound as compared to the monovalent disaccharide. The potency per sugar of the tetravalent compound was twice that of the octavalent compound. For *E. coli* expressing an adhesin belonging to the PapGI class, which recognizes terminal Galα1-4Gal, the inhibitory potency per sugar was highest for the octavalent compound, which suggests that the octavalent compound has more possibilities to interact with the tip-located fimbrial adhesins.

*S. suis* adhesion has been successfully inhibited with galabiose-containing oligosaccharide derivatives, and SadP adhesin is thus a promising target for anti-adhesion therapy. Derivatives of galabiose, as well as polyvalent galabiose dendrimers can inhibit adhesion of *S. suis* in the nanomolar range. These results suggest that anti-adhesion therapy could be a potential alternative to antibiotics in the treatment of *S. suis* infections.

**Table 3:** Inhibitory potencies of galabiose dendrimers against *S. suis* and *E. coli* adhesion (modified from [98]).

Bacterial strain and inhibitor	Valency of dendrimer	Relative potency	Potency per sugar
*S. suis* 628 ^a^			
Monovalent	1	1	1
Divalent, short spacer	2	13	7
Divalent, long spacer	2	12	8
Tetravalent	4	250	63
Octavalent	8	310	39
Octavalent PAMAM	8	260	32
*S. suis* D282 ^b^			
Monovalent	1	1	1
Divalent	2	50	25
Tetravalent	4	170	42
Tetravalent galatriose ^c^	4	8	2
Octavalent	8	100	13
*E. coli* PapG_J96_^a^			
Monovalent	1	1	1
Divalent, short spacer arms	2	3	1
Divalent, long spacer arms	2	3	2
Tetravalent	4	8	2
Octavalent	8	43	5
Octavalent PAMAM	8	6	1

**^a^** The IC_50_ values were determined in a surface plasmon resonance (SPR) assay [97]; relative potency = IC_50_ or the MIC of the monovalent inhibitor divided by that of the inhibitor in question; relative potency per sugar = relative potency/valency; ^b^ MIC values were determined in a hemagglutination assay [99]; ^c^ other inhibitors were galabiose derivatives.

## References

[1] Higgins R., Gottschalk M., Straw B.E., Zimmerman J.J., D’Allaire S., Taylor D.J. (2005). *Streptococcal* *diseases*. Diseases of Swine.

[2] Wertheim H.F., Nghia H.D., Taylor W., Schultsz C. (2009). *Streptococcus suis*: An emerging human pathogen. Clin. Infect. Dis..

[3] Feng Y., Zhang H., Ma Y., Gao G.F. (2010). Uncovering newly emerging variants of *Streptococcus suis*, an important zoonotic agent. Trends Microbiol..

[4] Mai N.T., Hoa N.T., Nga T.V., Linh le D., Chau T.T., Sinh D.X., Phu N.H., Chuong L.V., Diep T.S., Campbell J. (2008). *Streptococcus suis* meningitis in adults in Vietnam. Clin. Infect. Dis..

[5] Ye C., Zheng H., Zhang J., Jing H., Wang L., Xiong Y., Wang W., Zhou Z., Sun Q., Luo X. (2009). Clinical, experimental, and genomic differences between intermediately pathogenic, highly pathogenic, and epidemic *Streptococcus suis*. J. Infect. Dis..

[6] Pizarro-Cerda J., Cossart P. (2006). Bacterial adhesion and entry into host cells. Cell.

[7] Sharon N., Ofek I. (2000). Safe as mother’s milk: Carbohydrates as future anti-adhesion drugs for bacterial diseases. Glycoconj. J..

[8] Moschioni M., Pansegrau W., Barocchi M.A. (2010). Adhesion determinants of the *Streptococcus* species. Microb. Biotechnol..

[9] Holden M.T., Hauser H., Sanders M., Ngo T.H., Cherevach I., Cronin A., Goodhead I., Mungall K., Quail M.A., Price C. (2009). Rapid evolution of virulence and drug resistance in the emerging zoonotic pathogen *Streptococcus suis*. PLoS One.

[10] Ofek I., Hasty D.L., Sharon N. (2003). Anti-adhesion therapy of bacterial diseases: Prospects and problems. FEMS Immunol. Med. Microbiol..

[11] Sharon N. (2006). Carbohydrates as future anti-adhesion drugs for infectious diseases. Biochim. Biophys. Acta.

[12] Gottschalk M., Segura M., Xu J. (2007). *Streptococcus suis* infections in humans: The Chinese experience and the situation in North America. Anim. Health. Res. Rev..

[13] Fittipaldi N., Segura M., Grenier D., Gottschalk M. (2012). Virulence factors involved in the pathogenesis of the infection caused by the swine pathogen and zoonotic agent *Streptococcus suis*. Future Microbiol..

[14] Lalonde M., Segura M., Lacouture S., Gottschalk M. (2000). Interactions between *Streptococcus suis* serotype 2 and different epithelial cell lines. Microbiology.

[15] Benga L., Goethe R., Rohde M., Valentin-Weigand P. (2004). Non-encapsulated strains reveal novel insights in invasion and survival of *Streptococcus suis* in epithelial cells. Cell. Microbiol..

[16] Vanier G., Segura M., Friedl P., Lacouture S., Gottschalk M. (2004). Invasion of porcine brain microvascular endothelial cells by *Streptococcus suis* serotype 2. Infect. Immun..

[17] Tenenbaum T., Papandreou T., Gellrich D., Friedrichs U., Seibt A., Adam R., Wewer C., Galla H.J., Schwerk C., Schroten H. (2009). Polar bacterial invasion and translocation of *Streptococcus suis* across the blood-cerebrospinal fluid barrier *in vitro*. Cell. Microbiol..

[18] Schwerk C., Papandreou T., Schuhmann D., Nickol L., Borkowski J., Steinmann U., Quednau N., Stump C., Weiss C., Berger J. (2012). Polar invasion and translocation of *Neisseria meningitidis* and *Streptococcus suis* in a novel human model of the blood-cerebrospinal fluid barrier. PLoS One.

[19] Jobin M.C., Fortin J., Willson P.J., Gottschalk M., Grenier D. (2005). Acquisition of plasmin activity and induction of arachidonic acid release by *Streptococcus suis* in contact with human brain microvascular endothelial cells. FEMS Microbiol. Lett..

[20] Jobin M.C., Gottschalk M., Grenier D. (2006). Upregulation of prostaglandin E2 and matrix metalloproteinase 9 production by human macrophage-like cells: Synergistic effect of capsular material and cell wall from *Streptococcus suis*. Microb. Pathog..

[21] Pestova E.V., Havarstein L.S., Morrison D.A. (1996). Regulation of competence for genetic transformation in *Streptococcus pneumoniae* by an auto-induced peptide pheromone and a two-component regulatory system. Mol. Microbiol..

[22] Alloing G., Granadel C., Morrison D.A., Claverys J.P. (1996). Competence pheromone, oligopeptide permease, and induction of competence in Streptococcus pneumoniae. Mol. Microbiol..

[23] West A.H., Stock A.M. (2001). Histidine kinases and response regulator proteins in two-component signaling systems. Trends Biochem. Sci..

[24] Li J., Tan C., Zhou Y., Fu S., Hu L., Hu J., Chen H., Bei W. (2011). The two-component regulatory system CiaRH contributes to the virulence of *Streptococcus suis* 2. Vet. Microbiol..

[25] De Greeff A., Buys H., van Alphen L., Smith H.E. (2002). Response regulator important in pathogenesis of *Streptococcus suis* serotype 2. Microb. Pathog..

[26] Wu T., Chang H., Tan C., Bei W., Chen H. (2009). The orphan response regulator RevSC21 controls the attachment of *Streptococcus suis* serotype-2 to human laryngeal epithelial cells and the expression of virulence genes. FEMS Microbiol. Lett..

[27] Li M., Wang C., Feng Y., Pan X., Cheng G., Wang J., Ge J., Zheng F., Cao M., Dong Y. (2008). SalK/SalR, a two-component signal transduction system, is essential for full virulence of highly invasive *Streptococcus suis* serotype 2. PLoS One.

[28] Shen X., Zhong Q., Zhao Y., Yin S., Chen T., Hu F., Li M. (2013). Proteome Analysis of the Two-Component SalK/SalR System in Epidemic *Streptococcus suis* Serotype 2. Curr. Microbiol..

[29] Pan X., Ge J., Li M., Wu B., Wang C., Wang J., Feng Y., Yin Z., Zheng F., Cheng G. (2009). The orphan response regulator CovR: A globally negative modulator of virulence in *Streptococcus suis* serotype 2. J. Bacteriol..

[30] Anbalagan S., McShan W.M., Dunman P.M., Chaussee M.S. (2011). Identification of Rgg binding sites in the *Streptococcus pyogenes* chromosome. J. Bacteriol..

[31] Anbalagan S., Dmitriev A., McShan W.M., Dunman P.M., Chaussee M.S. (2012). Growth phase-dependent modulation of Rgg binding specificity in *Streptococcus pyogenes*. J. Bacteriol..

[32] Pulliainen A.T., Hytonen J., Haataja S., Finne J. (2008). Deficiency of the Rgg regulator promotes H2O2 resistance, AhpCF-mediated H2O2 decomposition, and virulence in *Streptococcus pyogenes*. J. Bacteriol..

[33] Hytonen J., Haataja S., Finne J. (2006). Use of flow cytometry for the adhesion analysis of *Streptococcus pyogenes* mutant strains to epithelial cells: Investigation of the possible role of surface pullulanase and cysteine protease, and the transcriptional regulator Rgg. BMC Microbiol..

[34] Zheng F., Ji H., Cao M., Wang C., Feng Y., Li M., Pan X., Wang J., Qin Y., Hu F. (2011). Contribution of the Rgg transcription regulator to metabolism and virulence of *Streptococcus suis* serotype 2. Infect. Immun..

[35] National Center for Biotechnology. http://www.ncbi.nlm.nih.gov/gene/.

[36] Wang Y., Zhang W., Wu Z., Zhu X., Lu C. (2011). Functional analysis of luxS in *Streptococcus suis* reveals a key role in biofilm formation and virulence. Vet. Microbiol..

[37] Willenborg J., Fulde M., de Greeff A., Rohde M., Smith H.E., Valentin-Weigand P., Goethe R. (2011). Role of glucose and CcpA in capsule expression and virulence of *Streptococcus suis*. Microbiology.

[38] Smith H.E., Damman M., van der Velde J., Wagenaar F., Wisselink H.J., Stockhofe-Zurwieden N., Smits M.A. (1999). Identification and characterization of the cps locus of *Streptococcus suis* serotype 2: The capsule protects against phagocytosis and is an important virulence factor. Infect. Immun..

[39] Feng Y., Cao M., Shi J., Zhang H., Hu D., Zhu J., Zhang X., Geng M., Zheng F., Pan X. (2012). Attenuation of *Streptococcus suis* virulence by the alteration of bacterial surface architecture. Sci. Rep..

[40] Fittipaldi N., Sekizaki T., Takamatsu D., Harel J., Dominguez-Punaro Mde L., von Aulock S., Draing C., Marois C., Kobisch M., Gottschalk M. (2008). D-alanylation of lipoteichoic acid contributes to the virulence of *Streptococcus suis*. Infect. Immun..

[41] Fittipaldi N., Sekizaki T., Takamatsu D., de la Cruz Dominguez-Punaro M., Harel J., Bui N.K., Vollmer W., Gottschalk M. (2008). Significant contribution of the pgdA gene to the virulence of *Streptococcus suis*. Mol. Microbiol..

[42] Ge J., Feng Y., Ji H., Zhang H., Zheng F., Wang C., Yin Z., Pan X., Tang J. (2009). Inactivation of dipeptidyl peptidase IV attenuates the virulence of *Streptococcus suis* serotype 2 that causes streptococcal toxic shock syndrome. Curr. Microbiol..

[43] Esgleas M., Dominguez-Punaro Mde L., Li Y., Harel J., Dubreuil J.D., Gottschalk M. (2009). Immunization with SsEno fails to protect mice against challenge with *Streptococcus suis* serotype 2. FEMS Microbiol. Lett..

[44] Feng Y., Pan X., Sun W., Wang C., Zhang H., Li X., Ma Y., Shao Z., Ge J., Zheng F. (2009). *Streptococcus suis* enolase functions as a protective antigen displayed on the bacterial cell surface. J. Infect. Dis..

[45] Brassard J., Gottschalk M., Quessy S. (2004). Cloning and purification of the *Streptococcus suis* serotype 2 glyceraldehyde-3-phosphate dehydrogenase and its involvement as an adhesin. Vet. Microbiol..

[46] Tan C., Fu S., Liu M., Jin M., Liu J., Bei W., Chen H. (2008). Cloning, expression and characterization of a cell wall surface protein, 6-phosphogluconate-dehydrogenase, of *Streptococcus suis* serotype 2. Vet. Microbiol..

[47] Si Y., Yuan F., Chang H., Liu X., Li H., Cai K., Xu Z., Huang Q., Bei W., Chen H. (2009). Contribution of glutamine synthetase to the virulence of *Streptococcus suis* serotype 2. Vet. Microbiol..

[48] Ju C.X., Gu H.W., Lu C.P. (2012). Characterization and functional analysis of atl, a novel gene encoding autolysin in *Streptococcus suis*. J. Bacteriol..

[49] De Greeff A., Buys H., Verhaar R., Dijkstra J., van Alphen L., Smith H.E. (2002). Contribution of fibronectin-binding protein to pathogenesis of *Streptococcus suis* serotype 2. Infect. Immun..

[50] Vanier G., Sekizaki T., Dominguez-Punaro M.C., Esgleas M., Osaki M., Takamatsu D., Segura M., Gottschalk M. (2008). Disruption of srtA gene in *Streptococcus suis* results in decreased interactions with endothelial cells and extracellular matrix proteins. Vet. Microbiol..

[51] Zhang A., Mu X., Chen B., Liu C., Han L., Chen H., Jin M. (2010). Identification and characterization of IgA1 protease from *Streptococcus suis*. Vet. Microbiol..

[52] Zhang A., Mu X., Chen B., Han L., Chen H., Jin M. (2011). IgA1 protease contributes to the virulence of *Streptococcus suis*. Vet. Microbiol..

[53] Baums C.G., Kaim U., Fulde M., Ramachandran G., Goethe R., Valentin-Weigand P. (2006). Identification of a novel virulence determinant with serum opacification activity in *Streptococcus suis*. Infect. Immun..

[54] Hu Q., Liu P., Yu Z., Zhao G., Li J., Teng L., Zhou M., Bei W., Chen H., Jin M. (2010). Identification of a cell wall-associated subtilisin-like serine protease involved in the pathogenesis of *Streptococcus suis* serotype 2. Microb. Pathog..

[55] Bonifait L., Grenier D. (2011). The SspA subtilisin-like protease of *Streptococcus suis* triggers a pro-inflammatory response in macrophages through a non-proteolytic mechanism. BMC Microbiol..

[56] Li W., Wan Y., Tao Z., Chen H., Zhou R. (2013). A novel fibronectin-binding protein of *Streptococcus suis* serotype 2 contributes to epithelial cell invasion and *in vivo* dissemination. Vet. Microbiol..

[57] Stroeher U.H., Paton A.W., Ogunniyi A.D., Paton J.C. (2003). Mutation of luxS of *Streptococcus pneumoniae* affects virulence in a mouse model. Infect. Immun..

[58] Vidal J.E., Howery K.E., Ludewick H.P., Nava P., Klugman K.P. (2013). Quorum sensing systems LuxS/AI-2 and Com regulate *Streptococcus pneumoniae* biofilms in a bioreactor with living cultures of human respiratory cells. Infect. Immun..

[59] Zhu H., Huang D., Zhang W., Wu Z., Lu Y., Jia H., Wang M., Lu C. (2011). The novel virulence-related gene stp of *Streptococcus suis* serotype 9 strain contributes to a significant reduction in mouse mortality. Microb. Pathog..

[60] Tikkanen K., Haataja S., Francois-Gerard C., Finne J. (1995). Purification of a galactosyl-alpha 1-4-galactose-binding adhesin from the Gram-positive meningitis-associated bacterium *Streptococcus suis*. J. Biol. Chem..

[61] Van Calsteren M.R., Gagnon F., Lacouture S., Fittipaldi N., Gottschalk M. (2010). Structure determination of *Streptococcus suis* serotype 2 capsular polysaccharide. Biochem. Cell Biol..

[62] Segura M., Gottschalk M. (2002). *Streptococcus suis* interactions with the murine macrophage cell line J774: Adhesion and cytotoxicity. Infect. Immun..

[63] Houde M., Gottschalk M., Gagnon F., van Calsteren M.R., Segura M. (2012). *Streptococcus suis* capsular polysaccharide inhibits phagocytosis through destabilization of lipid microdomains and prevents lactosylceramide-dependent recognition. Infect. Immun..

[64] Wewer C., Seibt A., Wolburg H., Greune L., Schmidt M.A., Berger J., Galla H.J., Quitsch U., Schwerk C., Schroten H. (2011). Transcellular migration of neutrophil granulocytes through the blood-cerebrospinal fluid barrier after infection with *Streptococcus suis*. J. Neuroinflammation.

[65] Fittipaldi N., Gottschalk M., Vanier G., Daigle F., Harel J. (2007). Use of selective capture of transcribed sequences to identify genes preferentially expressed by *Streptococcus suis* upon interaction with porcine brain microvascular endothelial cells. Appl. Environ. Microbiol..

[66] Nobbs A.H., Lamont R.J., Jenkinson H.F. (2009). Streptococcus adherence and colonization. Microbiol. Mol. Biol. Rev..

[67] Henderson B., Martin A. (2011). Bacterial virulence in the moonlight: Multitasking bacterial moonlighting proteins are virulence determinants in infectious disease. Infect. Immun..

[68] Singh B., Fleury C., Jalalvand F., Riesbeck K. (2012). Human pathogens utilize host extracellular matrix proteins laminin and collagen for adhesion and invasion of the host. FEMS Microbiol. Rev..

[69] Wang K., Lu C. (2007). Adhesion activity of glyceraldehyde-3-phosphate dehydrogenase in a Chinese *Streptococcus suis* type 2 strain. Berl. Munch. Tierarztl. Wochenschr..

[70] Wang C., Li M., Feng Y., Zheng F., Dong Y., Pan X., Cheng G., Dong R., Hu D., Feng X. (2009). The involvement of sortase A in high virulence of STSS-causing *Streptococcus suis* serotype 2. Arch. Microbiol..

[71] Baums C.G., Valentin-Weigand P. (2009). Surface-associated and secreted factors of *Streptococcus suis* in epidemiology, pathogenesis and vaccine development. Anim. Health. Res. Rev..

[72] Osaki M., Takamatsu D., Shimoji Y., Sekizaki T. (2002). Characterization of *Streptococcus suis* genes encoding proteins homologous to sortase of Gram-positive bacteria. J. Bacteriol..

[73] Takamatsu D., Nishino H., Ishiji T., Ishii J., Osaki M., Fittipaldi N., Gottschalk M., Tharavichitkul P., Takai S., Sekizaki T. (2009). Genetic organization and preferential distribution of putative pilus gene clusters in *Streptococcus suis*. Vet. Microbiol..

[74] Fittipaldi N., Takamatsu D., de la Cruz Dominguez-Punaro M., Lecours M.P., Montpetit D., Osaki M., Sekizaki T., Gottschalk M. (2010). Mutations in the gene encoding the ancillary pilin subunit of the *Streptococcus suis* srtF cluster result in pili formed by the major subunit only. PLoS One.

[75] Liukkonen J., Haataja S., Tikkanen K., Kelm S., Finne J. (1992). Identification of *N*-acetylneuraminyl alpha 2-->3 poly-*N*-acetyllactosamine glycans as the receptors of sialic acid-binding *Streptococcus suis* strains. J. Biol. Chem..

[76] Yuan Z.Z., Yan X.J., Zhang A.D., Chen B., Shen Y.Q., Jin M.L. (2013). Molecular mechanism by which surface antigen HP0197 mediates host cell attachment in the pathogenic bacteria *Streptococcus suis*. J. Biol. Chem..

[77] Haataja S., Tikkanen K., Nilsson U., Magnusson G., Karlsson K.A., Finne J. (1994). Oligosaccharide-receptor interaction of the Gal alpha 1-4Gal binding adhesin of *Streptococcus suis*. Combining site architecture and characterization of two variant adhesin specificities. J. Biol. Chem..

[78] Kouki A., Haataja S., Loimaranta V., Pulliainen A.T., Nilsson U.J., Finne J. (2011). Identification of a novel streptococcal adhesin P (SadP) recognizing galactosyl-{alpha}1-4-galactose-containing glycoconjugates: Convergent evolution of bacterial pathogens to binding of the same host receptor. J. Biol. Chem. J. Biol. Chem..

[79] Strömberg N., Marklund B.I., Lund B., Ilver D., Hamers A., Gaastra W., Karlsson K.A., Normark S. (1990). Host-specificity of uropathogenic *Escherichia coli* depends on differences in binding specificity to Gal alpha 1-4Gal-containing isoreceptors. EMBO J..

[80] Blanchard B., Nurisso A., Hollville E., Tetaud C., Wiels J., Pokorna M., Wimmerova M., Varrot A., Imberty A. (2008). Structural basis of the preferential binding for globo-series glycosphingolipids displayed by *Pseudomonas aeruginosa* lectin I. J. Mol. Biol..

[81] Lindberg A.A., Brown J.E., Strömberg N., Westling-Ryd M., Schultz J.E., Karlsson K.A. (1987). Identification of the carbohydrate receptor for Shiga toxin produced by *Shigella dysenteriae* type 1. J. Biol. Chem..

[82] Lingwood C.A., Law H., Richardson S., Petric M., Brunton J.L., de Grandis S., Karmali M. (1987). Glycolipid binding of purified and recombinant *Escherichia coli* produced verotoxin *in vitro*. J. Biol. Chem..

[83] Samuel J.E., Perera L.P., Ward S., O’Brien A.D., Ginsburg V., Krivan H.C. (1990). Comparison of the glycolipid receptor specificities of Shiga-like toxin type II and Shiga-like toxin type II variants. Infect. Immun..

[84] DeGrandis S., Law H., Brunton J., Gyles C., Lingwood C.A. (1989). Globotetraosylceramide is recognized by the pig edema disease toxin. J. Biol. Chem..

[85] Francois-Gerard C., Gerday C., Beeley J.G. (1979). Turtle-dove ovomucoid, a glycoprotein proteinase inhibitor with P1-blood-group antigen activity. Biochem. J..

[86] Takahashi N., Khoo K.H., Suzuki N., Johnson J.R., Lee Y.C. (2001). *N*-glycan structures from the major glycoproteins of pigeon egg white: Predominance of terminal Galalpha(1)Gal. J. Biol. Chem..

[87] Pian Y., Gan S., Wang S., Guo J., Wang P., Zheng Y., Cai X., Jiang Y., Yuan Y. (2012). Fhb, a novel factor H-binding surface protein, contributes to the antiphagocytic ability and virulence of *Streptococcus suis*. Infect. Immun..

[88] Nilsson U., Striker R.T., Hultgren S.J., Magnusson G. (1996). PapG adhesin from *E. coli* J96 recognizes the same saccharide epitope when present on whole bacteria and as isolated protein. Bioorg. Med. Chem..

[89] Striker R., Nilsson U., Stonecipher A., Magnusson G., Hultgren S.J. (1995). Structural requirements for the glycolipid receptor of human uropathogenic *Escherichia coli*. Mol. Microbiol..

[90] Kihlberg J., Hultgren S., Normark S., Magnusson G. (1989). Probing of the combining site of the PapG adhesin of uropathogenic *Escherichia coli* bacteria by synthetic analogs of galabiose. J. Am. Chem. Soc..

[91] Dodson K.W., Pinkner J.S., Rose T., Magnusson G., Hultgren S.J., Waksman G. (2001). Structural basis of the interaction of the pyelonephritic *E. coli* adhesin to its human kidney receptor. Cell.

[92] Ohlsson J., Larsson A., Haataja S., Alajääski J., Stenlund P., Pinkner J.S., Hultgren S.J., Finne J., Kihlberg J., Nilsson U.J. (2005). Structure-activity relationships of galabioside derivatives as inhibitors of *E. coli* and *S. suis* adhesins: Nanomolar inhibitors of *S. suis* adhesins. Org. Biomol. Chem..

[93] Pieters R.J. (2007). Intervention with bacterial adhesion by multivalent carbohydrates. Med. Res. Rev..

[94] Oberg C.T., Leffler H., Nilsson U.J. (2011). Inhibition of galectins with small molecules. Chimia (Aarau).

[95] Kitov P.I., Sadowska J.M., Mulvey G., Armstrong G.D., Ling H., Pannu N.S., Read R.J., Bundle D.R. (2000). Shiga-like toxins are neutralized by tailored multivalent carbohydrate ligands. Nature.

[96] Hansen H.C., Haataja S., Finne J., Magnusson G. (1997). Di-, tri-, and tetravalent dendritic galabiosides that inhibit hemagglutination by *Streptococcus suis* at nanomolar concentration. J. Am. Chem. Soc..

[97] Salminen A., Loimaranta V., Joosten J.A., Khan A.S., Hacker J., Pieters R.J., Finne J. (2007). Inhibition of P-fimbriated *Escherichia coli* adhesion by multivalent galabiose derivatives studied by a live-bacteria application of surface plasmon resonance. J. Antimicrob. Chemother..

[98] Kouki A. (2012). Identification and Characterization of a Novel Adhesin of *Streptococcus suis* and its Use as a Target of Adhesion Inhibition and Bacterial Detection. Ph.D. Thesis.

[99] Branderhorst H.M., Kooij R., Salminen A., Jongeneel L.H., Arnusch C.J., Liskamp R.M., Finne J., Pieters R.J. (2008). Synthesis of multivalent *Streptococcus suis* adhesion inhibitors by enzymatic cleavage of polygalacturonic acid and ‘click’ conjugation. Org. Biomol. Chem..

